# Inferring Polymorphism-Induced Regulatory Gene Networks Active in Human Lymphocyte Cell Lines by Weighted Linear Mixed Model Analysis of Multiple RNA-Seq Datasets

**DOI:** 10.1371/journal.pone.0078868

**Published:** 2013-10-30

**Authors:** Wensheng Zhang, Andrea Edwards, Erik K. Flemington, Kun Zhang

**Affiliations:** 1 Department of Computer Science, Xavier University of Louisiana, New Orleans, Louisiana, United States of America; 2 Department of Pathology, Tulane University Health Sciences Center and Tulane Cancer Center, New Orleans, Louisiana, United States of America; Semmelweis University, Hungary

## Abstract

Single-nucleotide polymorphisms (SNPs) contribute to the between-individual expression variation of many genes. A regulatory (trait-associated) SNP is usually located near or within a (host) gene, possibly influencing the gene’s transcription or/and post-transcriptional modification. But its targets may also include genes that are physically farther away from it. A heuristic explanation of such multiple-target interferences is that the host gene transfers the SNP genotypic effects to the distant gene(s) by a transcriptional or signaling cascade. These connections between the host genes (regulators) and the distant genes (targets) make the genetic analysis of gene expression traits a promising approach for identifying unknown regulatory relationships. In this study, through a mixed model analysis of multi-source digital expression profiling for 140 human lymphocyte cell lines (LCLs) and the genotypes distributed by the international HapMap project, we identified 45 thousands of potential SNP-induced regulatory relationships among genes (the significance level for the underlying associations between expression traits and SNP genotypes was set at FDR < 0.01). We grouped the identified relationships into four classes (paradigms) according to the two different mechanisms by which the regulatory SNPs affect their cis- and trans- regulated genes, modifying mRNA level or altering transcript splicing patterns. We further organized the relationships in each class into a set of network modules with the cis- regulated genes as hubs. We found that the target genes in a network module were often characterized by significant functional similarity, and the distributions of the target genes in three out of the four networks roughly resemble a power-law, a typical pattern of gene networks obtained from mutation experiments. By two case studies, we also demonstrated that significant biological insights can be inferred from the identified network modules.

## Introduction

Single-nucleotide polymorphisms (SNPs) represent the most abundant form (∼90%) of variation in the human genome. Genome-wide association studies have identified numerous phenotype-associated SNPs [1], [2], [3]. Recent studies showed that SNPs are predominant compared to copy number variations (CNV) in explaining the between-individual expression and splicing variation of many genes; and many of them are related to human diseases [4], [5], [6], [7], [8], [9], [10]. A regulatory (trait-associated) SNP is usually located near or within a host gene [6], [10], [11], [12], [13], possibly influencing the gene’s transcription or/and post-transcriptional modification. Its targets, besides the host gene, often include gene(s) physically farther away from it [9]. To date, several attempts have been made to explore the biological implications of such multiple-target interferences [9], [14], [15]. A heuristic explanation is that the host gene may transfer the SNP genotypic effects to the distant gene(s) by a transcriptional or signaling cascade [14]. This type of connections between the host genes (regulators) and the distant genes (targets) make the genetic analysis of gene expression traits a promising approach for identifying unknown regulatory relationships [9]. The mutation-mediated gene networks (modules) established in this way are highly valuable for understanding the mechanisms underlying the natural variation of complex traits and the development processes of genetic diseases [16], a central goal of medical genetics and personal medicine.

The primary task for inferring polymorphism-induced (-mediated) gene networks is to identify expression Quantitative Trait Locus (eQTL) and splicing Quantitative Trait Locus (sQTL) SNPs. The involved data collection process is usually time- and cost- demanding but has been greatly facilitated by the high throughput genomic technologies developed in the past years. In the HapMap project [17], millions of SNP loci of over a thousand lymphocyte cell lines (LCLs), each corresponding to an individual, have been genotyped. Several gene expression datasets of these samples generated on microarray or RNA-seq platforms have been deposited in the public databases such as GEO [18], SRA [19] and ArrayExpress [20]. However, the published results [7], [8], [9], each based on the computational analysis of a single dataset, are potentially subject to low statistical reliability and power due to the limited sample sizes. Presumably, more effective identification of the intrinsic associations between SNPs and expression traits can be achieved by an integrated joint analysis of these data using appropriate statistical methods. In this study, through a mixed model analysis of four RNA-seq datasets of the HapMap LCLs, we identified thousands of eQTL (sQTL) SNPs and, more importantly, the potential SNP-induced regulatory relationships active in normal immune cells. Two case studies on the established network modules with IQGAP1 and PKHD1L1 as hub genes further demonstrated that meaningful biological insights can be derived from these relationships.

## Results

**Figure 1:**
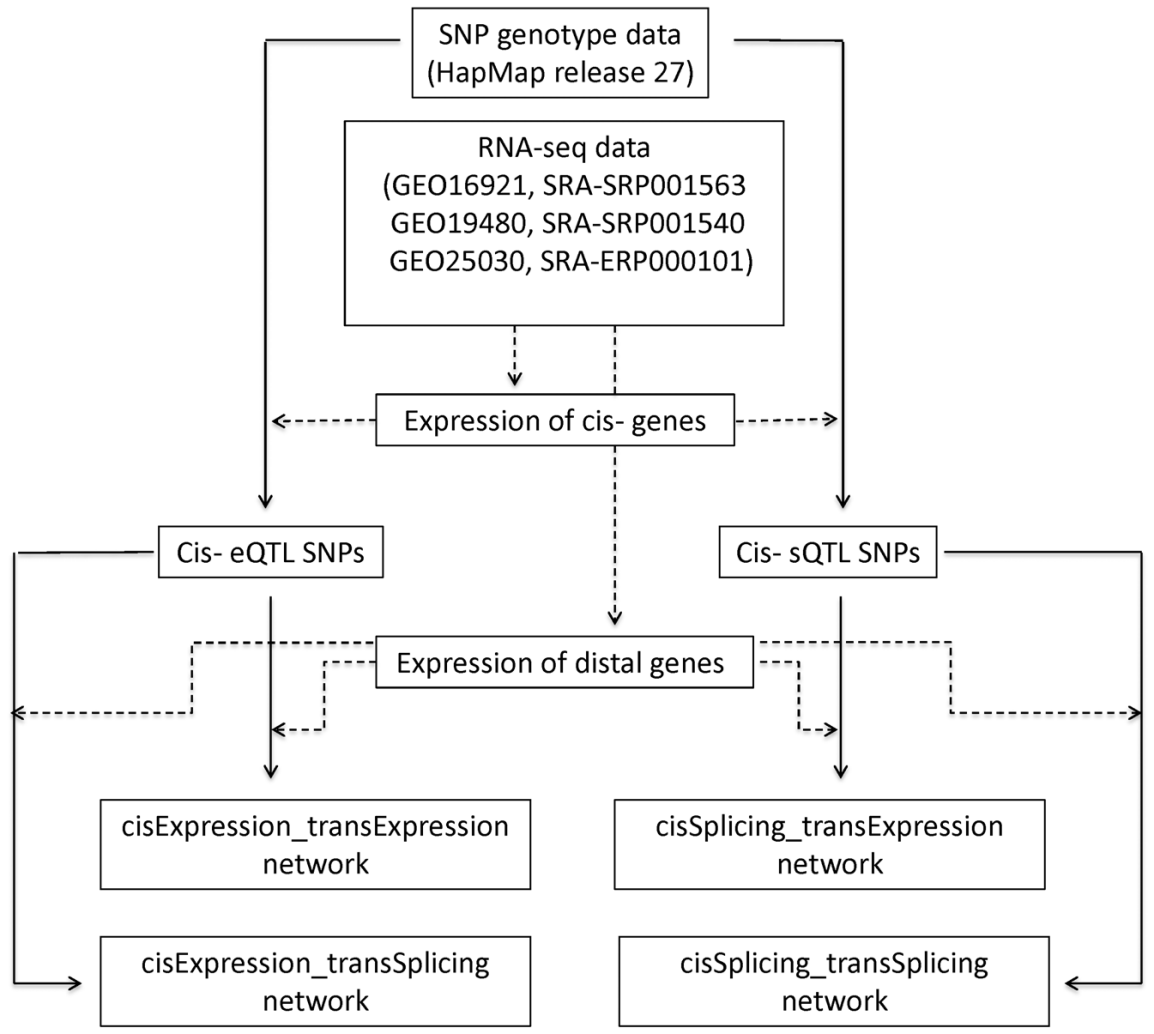
The schematic presentation of the study flow.

**Figure 1** presents the scheme of our study flow. The details of each step are described in this section and the Method section.

We collected RNA-seq data [7], [8], [9], deposited in NCBI SRA, for 261 biological samples of 140 human lymphocyte cell lines. Gene- and exon-level digital expression was inferred by the approaches described in the Method section. Based on these expression profiling and genotypes distributed by the HapMap Project (release 27), we identified the potential SNP-induced regulatory relationships among genes. For clarity purposes, hereafter, we use the phrase “cis-located” to indicate that a regulatory SNP is located in a gene or its extended sequences (20K *nt* upstream and 20K *nt* downstream).We use the term “regulation (regulated)” to denote the association between a SNP’s genotypes and a gene expression trait. We assumed that the regulatory relationship is “direct” when the SNP and the gene are cis- located to each other. This assumption is reasonable because the causal genetic element underlying the gene expression variance is likely the SNP itself or another DNA polymorphism that is in strong linkage disequilibrium with the SNP.

### Weighted linear mixed model and model evaluation

**Figure 2:**
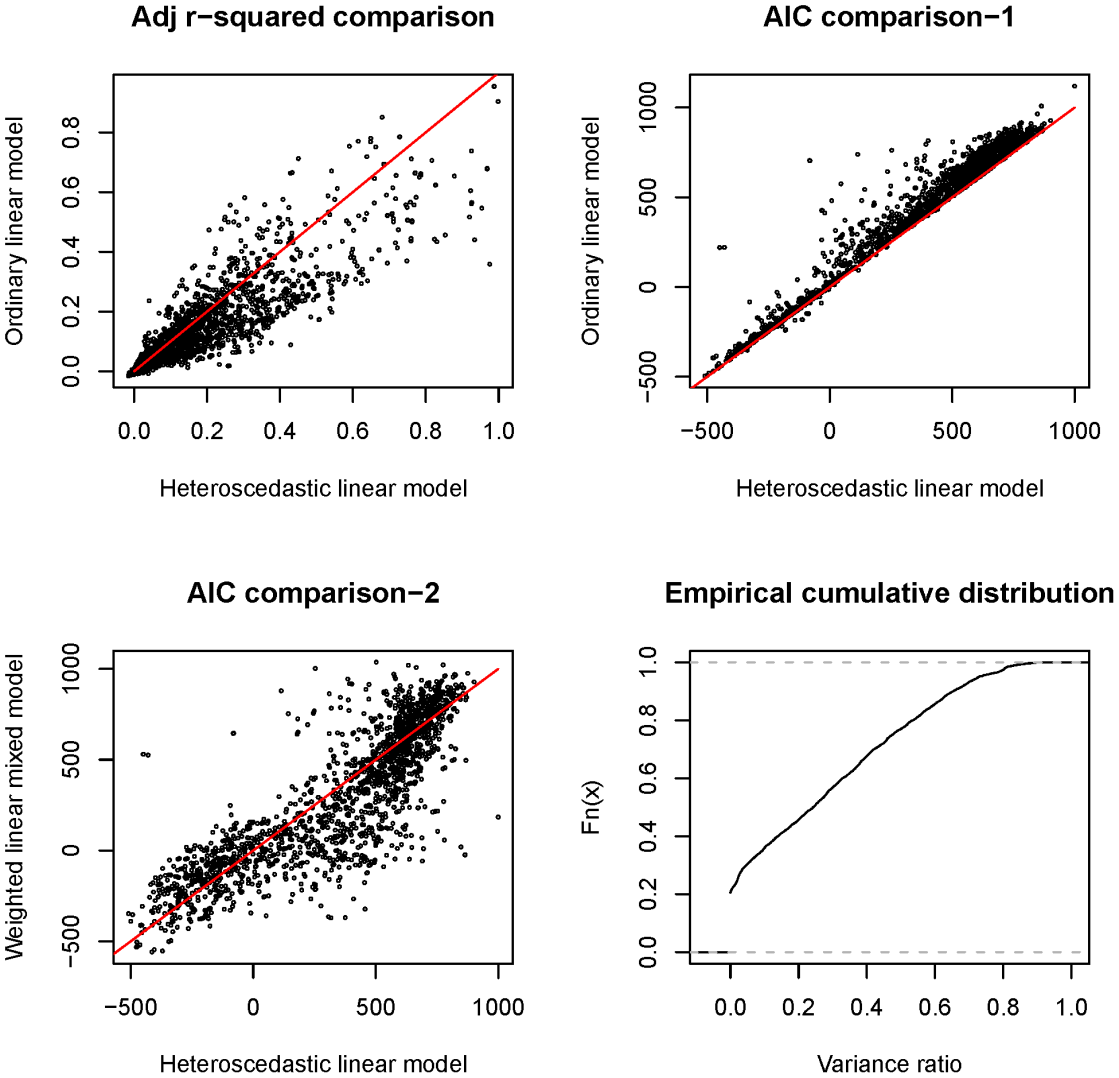
Model comparison and variance ratio distribution. In the scatter charts (plots **A**, **B** and **C**), each point represents a gene::SNP pair with the two Adj R-square (or AIC) values obtained by implementing the corresponding statistical models as specified by the x- and y- axis labels. In plot **D**, the variance ratio represents the proportion of the total variance accounted by the random effect component (SUBJECT) as estimated by the weighted linear mixed model.

In this study, the analyzed RNA-seq datasets were generated by four laboratories (**Table 1**). The tested cell lines (individuals) were sourced from two populations, i.e. CEU (US residents of Northern and Western European descent) and YRI (Ibadan, Nigeria). Around 70% of the individuals were measured two or three times. Therefore, the sequence depth, read length and thus the variability of the inferred expression levels were quite different from one dataset to another. Considering these complexities, i.e. the variance heterogeneity and batch effects of expression traits across multiple datasets as well as the dependence between the multiple measurements of the same individual, we conducted the association analysis by implementing a pair of weighted linear mixed (effects) models (WLMM). One model (Model-1a) was for SNP genotypes and gene expression association analysis and thus facilitates the identification of cis eQTL SNPs. The other model (Model-2a) was for SNP genotypes and gene transcript splicing association analysis and whereby enabling the detection of cis sQTL SNPs (see the Method section for details). In the models, GROUP (representing the batches associated with laboratories and populations) and SUBJECT (representing cell lines) were included as the fixed effect factor and random effect factor, respectively. However, the inclusion of additional parameters in the mixed models over an ordinary linear model (OLM, such as Model-1c) or a heteroscedastic linear (fixed effects) model (HLM, such as Model-1b) may introduce artificial noise. In this regard, we conducted a preliminary study to compare WLMM to HLM/OLM based on the adjusted R^2^ and AIC criteria. We focused the analysis on the association between the expression levels of the genes on chromosome one and the genotypes of ∼8000 pruned cis-located SNPs (see the Method section) using Models-1a, -1b and -1c. As shown in **Figure 2A-B**, HLM was generally superior to OLM with respect to the higher R^2^ and lower AIC values for most gene::SNP pairs. WLMM modestly outperformed HLM in terms of the lower AIC values for ∼55% of gene::SNP pairs (**Figure 2C**). The fitness of WLMM to our data was further verified by a variance component analysis, which showed that the proportion of the total variance accounted by the random factor was substantial (**Figure 2D**). R^2^ based comparison between WLMM and HLM is not presented here because, in statistics, the criterion is not recommended for evaluating a mixed model.

**Table 1:** Overview of the analyzed RNA-seq Data.

Group	SR	CL	RD	RL	Population	LAB	Reference	
A	41	41	38.82	50	CEU	U-Penn	Cheung et al, 2010 [9]	
B1	81	69	8.41	35	YRI	U-Yale	Pickrell et al, 2010 [8]	
B2	80	69	8.33	46	YRI	Argonne	Pickrell et al, 2010 [8]	
C	59	59	21.21	36	CEU	U-Geneva	Montgomery et al, 2010 [7]	

SR: The number of biological samples. CL: the number of HapMap cell lines. RD: the median of the numbers of mapped reads (in millions). RL: read length (*nt*). LAB: the institute or company that generated the data.

### Cis- eQTLs and sQTL SNPs

**Figure 3:**
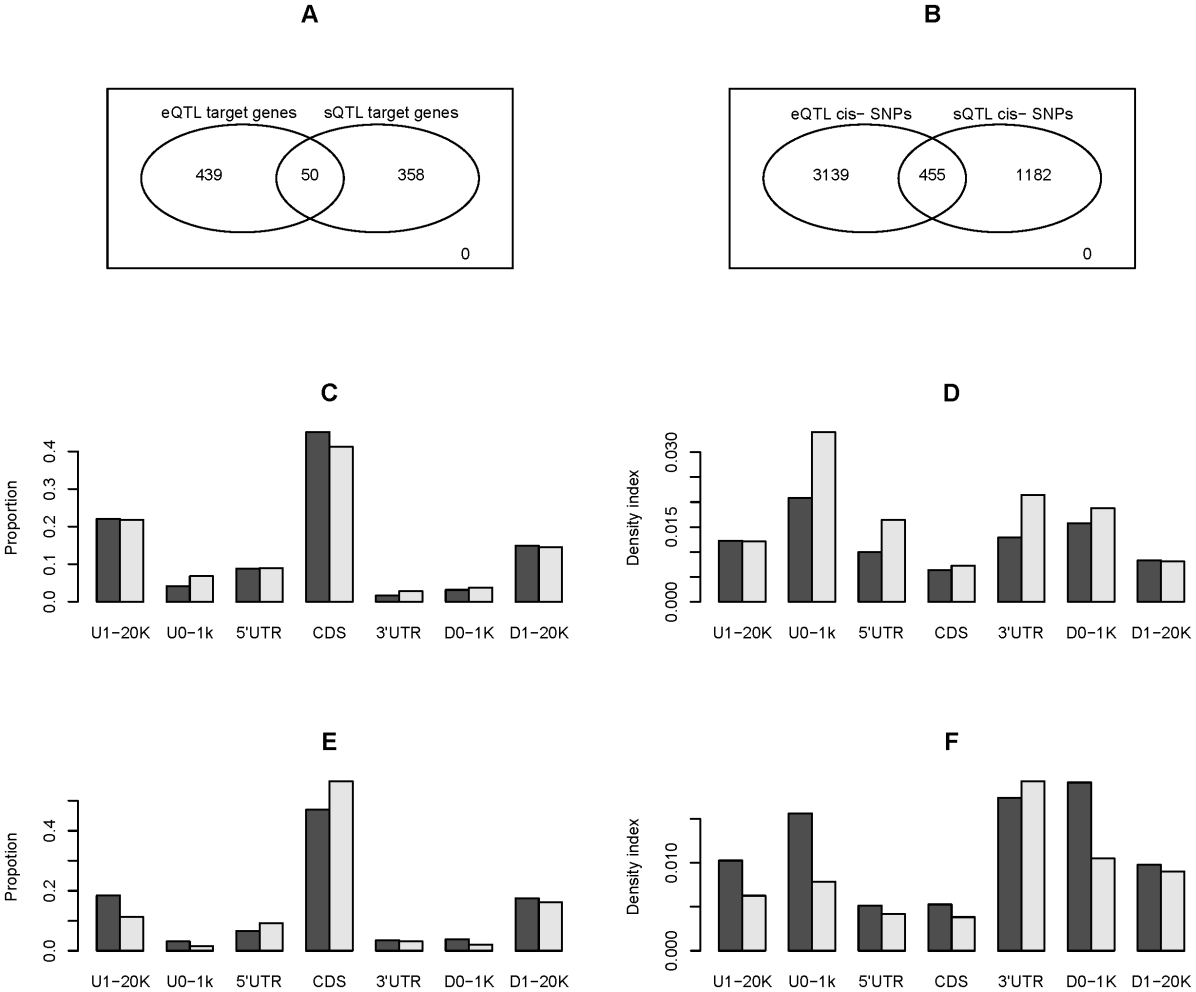
The distribution profiles of eQTL SNPs and sQTL SNPs across different genomic regions. In plot **A**, the result was summarized according to the involved genes (RefSeq mRNAs). In plot **B**, the result was summarized according to the involved SNPs. In the bar charts, the quantities for the entire set of the eQTL (sQTL) SNPs are represented by black bars and the quantities for the tag-SNPs (gene-wide most significant SNPs) are represented by grey bars. U0-1K/D0-1K represents the 0-1 kilo-bases upper-/down- stream region of a RefSeq gene and U1-20K/D1-20K represents the 1−20 kilo-bases upper-/down- stream region of a RefSeq gene. Plots **C**-**D** are drawn for eQTLs and Plots **E**-**F** are drawn for sQTLs. In plots **C** and **E**, “proportion” represents the ratio of the number of eQTL (sQTL) SNPs in the corresponding region to the total number of eQTL (sQTL) SNPs. In plots **D** and **F**, “density index” is calculated by dividing the proportion of eQTL (sQTL) SNPs with the average length (in kilo-base) of the corresponding genomic region.

We define an eQTL SNP as a polymorphism that meets the following two requirements. First, the SNP is either within a gene, up to 20Knt proximal to the start of the gene, or up to 20Knt distal to the end of the gene. Second, the genotypes of the SNP should be significantly associated with the expression level of the gene. Similarly, a sQTL SNP is defined as one that is either located within a gene, up to 20Knt proximal to the start of the gene, or up to 20Knt distal to the end of the gene, and whose genotypes is significantly associated with the transcript splicing of the gene. eQTLs SNPs were identified by Model-1a with the threshold set at FDR < 0.01 (ordinary p-value less than 8.0×10^−6^). sQTLs SNPs were detected by a two-step approach in order to take advantage of linear mixed models and ease the control of false discoveries due to the computational problem as discussed in the Method section. More specifically, a candidate list of SNP::exon associations was first generated by implementing Model-2a with threshold set at FDR < 0.05 (ordinary p-value less than 2.7×10^−9^). Then, this list was refined by Model-2b with threshold set at FDR < 0.01 (ordinary p-value less than 9.8×10^−9^). As shown in **Figure 3A and 3B**, we identified 3594 eQTL SNPs and 1637 sQTL SNPs with 455 SNPs being overlapped in those two sets, amounting to 12.7% of the former set or the 27.8% of the latter set. Those eQTLs (sQTL) SNPs are located in 489 (408) genes or their flanking sequences. Functional enrichment analysis showed that these genes had strong functional similarity. In particular, several gene ontology (GO) terms related to immune response were over-represented by two gene sets and a GO term related to mitochondrion was over-represented by the genes hosting the eQTL SNPs (**Tables 2** and **3**). The complete lists of the identified eQTL (sQTL) SNPs were summarized in **Tables S1-2**.

**Table 2:** Functional enrichment analysis of cis- located eQTL genes.

Term	Count	P-value	Fold Enrichment
GO:0005739(CC)∼mitochondrion	46	1.32E-06	2.14
GO:0019882(BP)∼antigen processing and presentation	9	1.85E-04	5.64
GO:0042613(CC)∼MHC class II protein complex	6	2.31E-04	10.45
GO:0002504(BP)∼antigen processing and presentation of peptide or polysaccharide antigen via MHC class II	6	3.79E-04	9.46
GO:0032395(MF)∼MHC class II receptor activity	5	4.21E-04	13.56
GO:0004364(MF)∼glutathione transferase activity	5	5.18E-04	12.88
GO:0016765(MF)∼transferase activity, transferring alkyl or aryl (other than methyl) groups	6	2.47E-03	6.31
GO:0048639(BP)∼positive regulation of developmental growth	4	3.23E-03	13.01
GO:0042611(CC)∼MHC protein complex	6	5.20E-03	5.32
GO:0002483(BP)∼antigen processing and presentation of endogenous peptide antigen	3	5.21E-03	26.02

**Table 3:** Functional enrichment analysis of cis- located sQTL genes.

Term	Count	P-value	Fold Enrichment
GO:0005938(CC)∼cell cortex	15	1.94E-09	8.58
GO:0043228(CC)∼non-membrane-bounded organelle	58	6.69E-07	1.87
GO:0005516(MF)∼calmodulin binding	12	1.30E-06	6.87
GO:0032395(MF)∼MHC class II receptor activity	6	2.78E-06	25.31
GO:0051015(MF)∼actin filament binding	8	3.78E-06	12.10
GO:0005856(CC)∼cytoskeleton	37	3.99E-06	2.24
GO:0008092(MF)∼cytoskeletal protein binding	21	4.21E-06	3.34
GO:0005829(CC)∼cytosol	36	4.56E-06	2.26
GO:0042611(CC)∼MHC protein complex	8	4.70E-06	11.73
GO:0019882(BP)∼antigen processing and presentation	9	7.84E-06	8.78

We characterized the distribution of the identified eQTL (sQTL) SNPs on their host genes over different genomic regions. We first divided the extended DNA sequence of a protein-coding gene cis- regulated by one (or multiple) eQTL (sQTL) SNP(s) into six regions: 1−20 kilo-bases upstream (U1-20K), 0−1 kilo-bases upstream (U0-1K), 5’UTR, coding region, 3’UTR, 0−1 kilo-bases downstream (D0-1K) and 1−20 kilo-bases downstream(D1-20K). Then, the identified eQTL (sQTL) SNPs were mapped on these regions. The results for 3123 eQTL SNPs on 426 coding genes and 1527 sQTL SNPs on 382 coding genes were summarized in **Figure 3C-F**. We found that, eQTL (sQTL) SNP density index (See the legend of **Figure 3** for the computation) in the coding regions was lower than that in the other regions, although ∼40% of eQTL (sQTL) SNPs were located in them. The promoter regions (represented by U0-1K) and 3’UTRs demonstrated the highest eQTL SNP and sQTL SNP density indexes, respectively. This tendency was more apparent when only the gene-wide most significant SNPs, similar to the tag-SNPs frequently called in literature [21], [22], were considered, as shown by the grey bars in the plots.

### SNP-induced regulatory gene networks

For each eQTL (sQTL) SNP, we scanned the association between its genotypes and the expression levels or transcript splicing measures of the genes that are either located on a different chromosome or on the same chromosome but with at least 1M *nt* distance. We use the phrase “trans- regulation (regulated)” to indicate a SNP::gene association of this type. We selected the significant association based on the same ordinary p-value thresholds used in identifying the eQTL (sQTL) SNPs. As a result, we detected forty five thousands potential SNP-induced regulatory relationships among genes. We further grouped the identified relationships into four classes, each of which represents a specific network paradigm, based on the mechanisms by which the regulatory SNPs affect their cis- and trans- regulated genes, modifying mRNA level or altering transcript splicing pattern. The four relationship classes (paradigms) were defined and explained as follows. Their compositions were summarized in **Table 4**.

**Table 4:** Summary of SNP induced gene networks.

Paradigms	# Regulator genes	# Target genes	# SNPs	# Connections
cisExpression_transExpression	283	1432	1051	2661
cisSplicing_transExpression	259	1397	609	3045
cisExpression_transSplicing	141	1237	494	6240
cisSplicing_transSplicing	241	4040	476	34244

*cisExpression_transExpression (C1):* Each connection in this class links a gene (regulator) whose expression level is cis- regulated by a regulatory SNP to another gene (target) whose expression level is trans- regulated by the same SNP.

*cisSplicing_transExpression (C2):* Each connection in this class links a gene (regulator) whose transcript splicing is cis- regulated by a regulatory SNP to another gene (target) whose expression level is trans- regulated by the same SNP.

*cisExpression_transSplicing (C3):* Each connection in this class links a gene (regulator) whose expression level is cis- regulated by a regulatory SNP to another gene (target) whose transcript splicing is trans- regulated by the same SNP.

*cisSplicing_transSplicing (C4):* Each connection in this class links a gene (regulator) whose transcript splicing is cis- regulated by a regulatory SNP to another gene (target) whose transcript splicing is trans- regulated by the same SNP.

*A remark on the paradigm definitions*: In C1-C4, a cis- regulated gene is called as a “regulator” of the cognate trans- regulated gene(s). Underlying this definition is our general assumption (see the Introduction section) for a mutation-mediated gene network. That is, the host gene of a SNP may transfer the genotypic effects to the distant gene(s) by a transcriptional or signaling cascade. The assumption is a direct extension of [14] where the focused genetic variations are artificially created.

**Figure 4:**
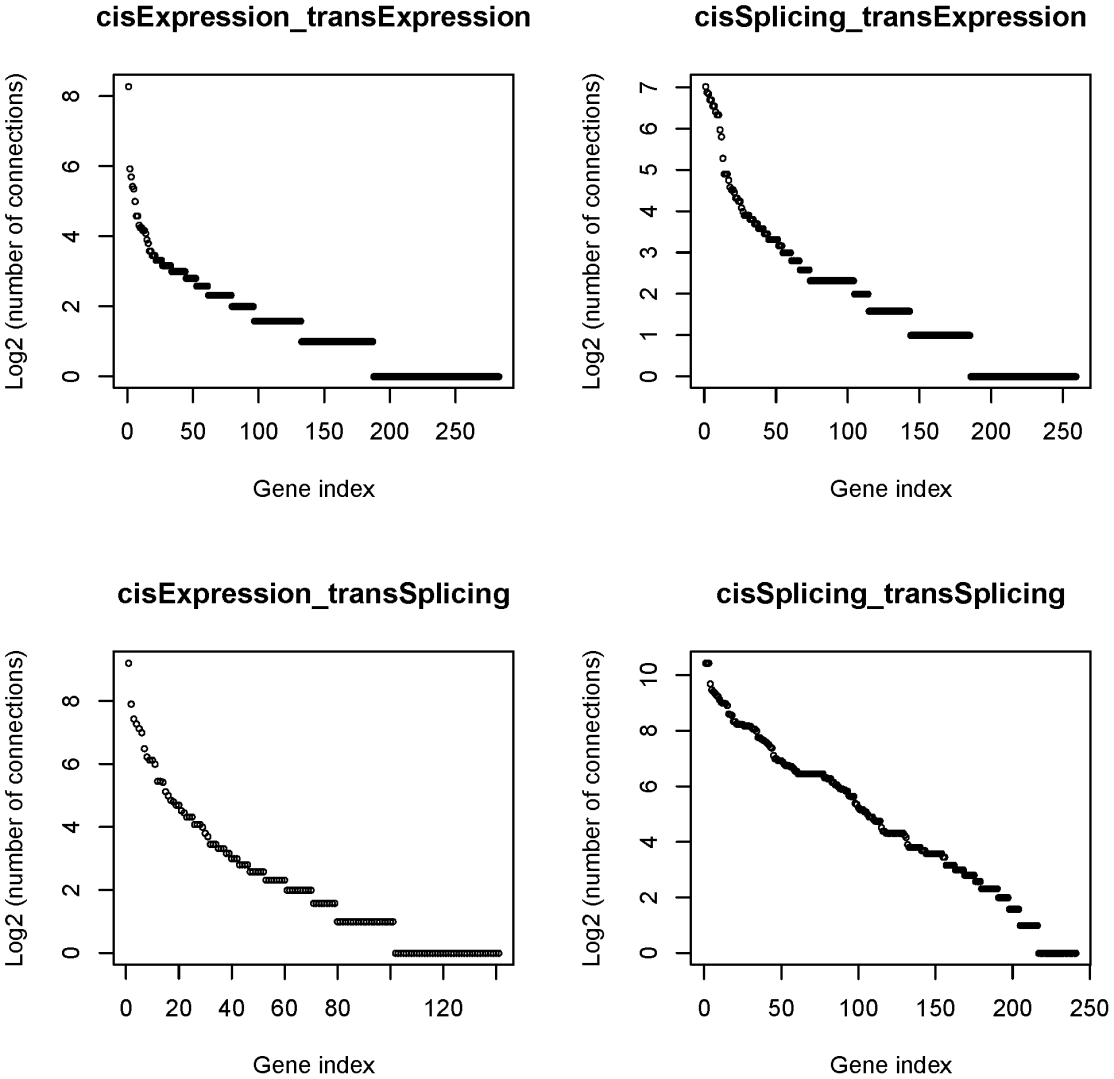
The uneven distributions of the regulated target genes. In drawing the plots, cis- located eQTL (sQTL) genes (regulators) are ordered by the numbers of their trans- located target genes and each of them is assigned an integer index (1, 2, 3,...).

We organized the relationships in each class into a set of network modules with the cis- regulated genes as the hubs. We visualized the distribution of the targets (trans- regulated genes) in each module in **Figure 4**. The profiles for C1, C2 and C3 roughly resembled a power-law distribution [23], i.e. most regulators (cis- regulated genes) had few targets, while few regulators had many. Such a resemblance is further suggested by the double logarithmic charts [24], [25] for the numbers of regulated target genes in these three relationship classes (**Figure S1**). C4 was particular in that about half of the regulators each had at least 20 targets. The top ten C1- and C2- types’ modules (in terms of the number of the trans- regulated genes) were presented in **Table 5**. The more comprehensive results can be found in **Tables S3-6**.

**Table 5:** Top SNP-induced gene network modules.

Gene ID^a^	Symbol	Disease gene^b^	# SNP	# Target genes	# Disease-related target genes
*cisExpression_transExpression*					
NM_017761	PNRC2		1	301	44
NM_177531	PKHD1L1		10	61	13
NM_032024	C10orf11		4	52	10
NM_052849	C15orf57		7	43	6
NR_024079	C2orf52		4	41	6
NM_001080792	C15orf57		6	32	3
NM_014435	NAAA		6	24	0
NM_152925	CPNE1		9	24	2
NM_016424	LUC7L3		1	20	2
NM_000255	MUT	x	6	19	4
*cisSplicing_transExpression*					
NM_006197	PCM1	x	6	130	16
NM_003171	SUPV3L1	x	1	118	23
NM_003870	IQGAP1		6	115	18
NM_004946	DOCK2	x	15	104	6
NM_015187	SEL1L3		1	104	9
NM_001199282/_007762	LRBA		20	94	20
NM_004481	GALNT2	x	1	85	5
NM_015601/_022079	HERC4		1	81	16
NM_002661	PLCG2		8	63	4
NM_014689	DOCK10		9	56	6

a NCBI RefSeq ID of the genes that are cis- located with eQTL (sQTL) SNPs. ^b^ x indicates the association between the gene and a disease has been reported in literature as collected by DAVID [28].

**Figure 5:**
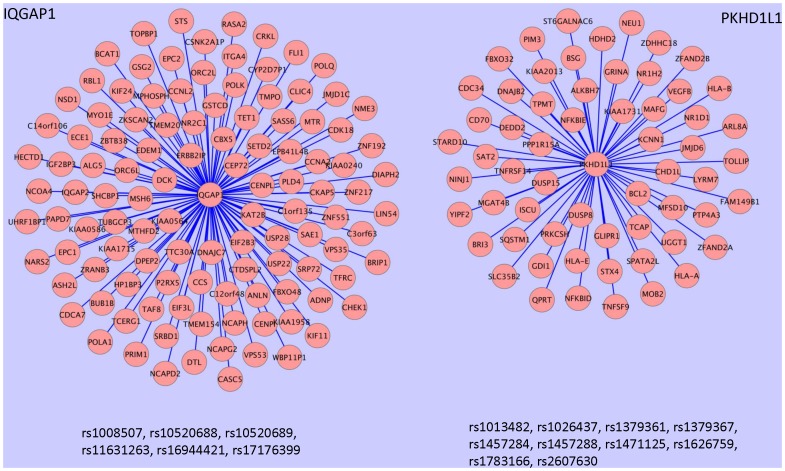
Case studies of polymorphism-induced gene regulation network and the biological implications. The sub-network (*cisSplicing_transExpression* paradigm) on the left shows that IQGAP1 gene, whose alternative splicing is associated with the genotypes of six SNPs, is a potential regulator of 115 trans- gene RefSeq genes at 113 loci, each of which has the expression level statistically affected by at least one SNP of the same set. These target genes are widely involved in cell cycle (see the left section of **Table 6**). The sub-network (*cisExpression_transExpression* paradigm) on the right shows that PKHD1L1 gene, whose expression level is associated with the genotypes of ten SNPs, is a potential regulator of 61 trans RefSeq genes at 60 loci, each of which has the expression level statistically affected by at least one SNP of the same set. A few immunity related GO terms are over-represented by these target genes as summerized in the right section of

We showcased the biological implications of the identified network modules for genes IQGAP1 and PKHD1L1 by **Figure 5** and **Table 6**., IQGAP1 gene encodes Ras GTPase-activating-like protein that is involved in the regulation of cell cycle and potentially plays a role in cancer [26], [27]. We found that the transcript splicing of the IQGAP1 gene was cis- regulated by genotypes of six SNPs and these sQTL SNPs also regulated the expression levels of other 115 genes physically far from them. An analysis using the David tool [28] showed that at least five cell cycle-related GO terms were over-represented by these trans-located genes. Hereby, the network module with IQGAP as the hub suggested the candidate mechanism (pathway) for its role in regulating cell cycle process. According to the DAVID database [28], [29], 16 targets in this module have been annotated as disease-related genes. For example, BRIP1 encodes Fanconi anemia group J protein, which appears to be important in ovarian cancer where it potentially act as an antioncogene [30]. As another example, MTR encodes methionine synthase that forms part of the S-adenosylmethionine (SAMe) biosynthesis and regeneration cycle [31]. Deficiency of methionine synthase can cause adult-onset leukoencephalopathy [32].

**Table 6:** Functional enrichment analysis of the target genes IQGAP1 and PKHD1L1.

IQGAP1			PKHD1L1		
Term a	PValue	Fold Enrichment	Term a	PValue	Fold Enrichment
GO:0005694(CC)∼chromosome	1.76E-10	7.25E+00	GO:0006955(BP)∼immune response	9.40E-04	4.20E+00
GO:0044427(CC)∼chromosomal part	1.25E-09	7.68E+00	GO:0002474(BP)∼antigen processing and presentation of peptide antigen via MHC class I	1.18E-03	5.68E+01
GO:0007049(BP)∼cell cycle	3.16E-08	4.17E+00	GO:0032393(MF)∼MHC class I receptor activity	1.35E-03	5.33E+01
GO:0022402(BP)∼cell cycle process	1.82E-07	4.68E+00	GO:0006915(BP)∼apoptosis	2.00E-03	4.28E+00
GO:0022403(BP)∼cell cycle phase	6.33E-07	5.33E+00	GO:0012501(BP)∼programmed cell death	2.18E-03	4.22E+00
GO:0051276(BP)∼chromosome organization	7.48E-07	4.85E+00	GO:0048002(BP)∼antigen processing and presentation of peptide antigen	3.22E-03	3.45E+01
GO:0000278(BP)∼mitotic cell cycle	1.08E-06	5.56E+00	GO:0042612(CC)∼MHC class I protein complex	3.26E-03	3.42E+01
GO:0000279(BP)∼M phase	1.98E-06	5.81E+00	GO:0008219(BP)∼cell death	5.38E-03	3.58E+00
GO:0000228(CC)∼nuclear chromosome	2.17E-06	1.03E+01	GO:0016265(BP)∼death	5.59E-03	3.56E+00
GO:0000087(BP)∼M phase of mitotic cell cycle	2.53E-06	7.22E+00	GO:0006470(BP)∼protein amino acid dephosphorylation	7.54E-03	9.69E+00

a Top 10 over-represented GO terms.

PKHD1L1 (polycystic kidney and hepatic disease 1 like 1) gene encodes a member of the polycystin protein family that may play a role in the male reproductive system [33]. A previous study reported that this gene was widely expressed at a low level in most tissues except blood-derived cell lines [34]. We found its expression level in LCLs was cis- regulated by the genotypes of ten SNPs and these eQTL SNPs also regulated the expression levels of other 63 genes physically far from them. As shown in the right section of **Table 6**, the potential role of PKHD1L1 gene in the regulation of immune response was suggested by the composition of the target genes. Importantly, the targets included three MHC (major histocompatibility complex) class I protein complex genes (HLA-A, HLA-B and HLA-E) that were not in the cis- located eQTL (sQTL) gene sets, indicating that the expression of MHC (HLA, the Human Leukocyte Antigen) genes can be trans- regulated by the mutations occurring in a genomic region distal from them. Another important gene in the PKHD1L1 module is BCL2. This gene encodes B-cell lymphoma 2 protein that regulates cell death (apoptosis) [35] and plays a critical role in the tumorigenesis of several cancers [36], [37].

## Discussion

In this study, we established a novel landscape of mutation-induced regulatory gene network paradigms and network modules active in normal human immune cells. Similar to a recent work [9], we defined a network connection by three elements, i.e. a regulatory SNP, a gene (regulator) whose sequence (or flanking sequences) contains the SNP, and a gene (target) whose expression or transcript splicing is trans- regulated by the SNP’s genotypes. However, our connection identification procedure is based on a prior assumption that the regulator gene transfers the SNP genotypic effects to the target gene by a transcriptional or signaling cascade. That is, for a SNP-mediated regulatory relationship between genes, we assume that the regulator itself has to be significantly associated with the SNP genotypes. This assumption was not used in [9] but it is critical for explaining the multiple-target interference of a functional mutation. Another highlight of our study is that, the identified network landscape is more comprehensive than the previous studies [9], [14] in that both mechanisms, modifying mRNA expression levels or altering transcript splicing patterns, by which the regulatory SNPs affect their cis- and trans- regulated genes are examined in a systematic way.

The findings in this study not only are valuable for understanding the mechanisms underlying the natural variation of complex traits but also can contribute to the functional annotation of the cognate genes. For example, PKHD1L1 has not been documented as an important disease gene. However, we noted that it potentially regulates three class I MHC genes and the oncogene BCL2 (**Figure 5**), implying its involvement in tumorigenesis and other disease process.

While the polymorphism-mediated regulatory relationships determined solely by computational methods should be regarded as candidate hypotheses to be tested, they are likely to be confirmed by biological validation due to the following observations. First, many of genes hosting the eQTL (sQTL) SNPs are involved in immunity, i.e. a few GO terms related to immune response are over-represented by both the gene sets. This functional enrichment is consistent with the nature of the tested cell lines and the well-known associations between mutations of MHC genes and disease resistance in humans and animals [38], [39]. Second, the distributions of regulated target genes in three (C1, C2 and C3) of the four SNP-mediated between-genes relationship classes identified by us roughly resemble a power-law, the typical pattern of gene networks obtained by mutation experiments [14], [40]. Third, the distribution profiles (**Figure 3D-F**) of eQTL SNPs and sQTL SNPs confirm the results of previous studies or biological intuitions, i.e. the former has a peak near 5’ ends of genes including transcription start sites (TSS) and promoter regions [8], [11], [12], and the later has a peak near 3’ ends of genes including 3’UTRs [6]. Lastly, as demonstrated in the case studies, the target genes in a network module are often characterized by the significant functional similarity. For example, in the modules with IQGAP as the hub, the biological role of the regulator can be well explained by the functions of the target genes. Our study is naturally a joint re-analysis of previously published datasets. A motivation of this effort is that we believe more general and unified results can be obtained from an integrative analysis of the multiple (expression) datasets with increased samples. We found that our conclusion regarding the association between the eQTL SNPs and immune response can be also derived from the results of [8]. However, compared to our finding, the significance of SNPs on the gene expression variance involved in longevity-related mitochondrial components [41], [42], a trait with middle-level heritability in human [43], was less remarkable in [8]. More specifically, as shown in **Table 2**, a gene ontology term (GO:0005739∼mitochondrion) in the cellular component category is over-represented by the 489 host genes of eQTL SNPs selected with FDR<0.01, and such a functional enrichment can be observed for the cis- eQTL gene set (n  =  929) identified in [8] with FDR<0.1 but not for the refined set (n  =  411) with FDR<0.01. We also noted that top gene regulatory modules (subnets) discovered in our study were not consistent with those identified in [9] where the network was defined similar to C1. At present, although we lack facilities to validate our results by biological experiments, we emphasize the suitability of the employed statistical methods for the analyzed data. The weighted linear mixed models not only can address the batch effects across different datasets and the dependence between the multiple measurements of the same individual, but also can achieve more dependable results on the dataset(s) with less random noise-sourced variability by assigning a larger weight to the samples in the set._Its advantages over linear fixed effects models have been showed in the Results section. Furthermore, it is worth noting that such variance heterogeneity and batch effects cannot be handled by the data preprocessing method used in [12], because the assumption that expression values across different datasets are from the same distributions can be spurious.

This study represents our initial attempt to explore polymorphism-mediated (induced) regulatory gene networks in human LCLs. We have identified three problems worth of further investigation. First, most of the detected regulator::target relationships are mediated by two or more regulatory SNPs that are often in strong linkage disequilibrium. This situation is further complicated by the SNPs that are not included in the HapMap project database but have been identified by the 1000 Genome Project [44]. If this is the case, for the identified relationships between two genes, the causal mutations [22], [45] still remain to be elucidated. Second, while we included all available RNA-seq datasets of HapMap LCLs when we started this study, approximately 40% of the individuals in the analyzed data did not have any biological replications. The data sparsity caused by this issue can greatly compromise the advantage of a mixed model in identifying the associations between gene expression measures and SNP genotypes, thus frequently leads to computational intractability. On the other hand, several microarray gene expression data sets for HapMap samples have been published [4], [5], [6], [12], [46]. Hereby, we believe that the problem arising from the insufficiency of available expression profiles for the genotyped LCLs can be alleviated by a more comprehensive joint analysis of RNA-seq data and microarray data. The third issue arises from the comparison of the identified between-genes relationship classes (C1, C2, C3 and C4). In C4, each regulator gene is connected to 16.7 target genes on average, roughly two times higher than those regulator genes in other three classes. Therefore, we hypothesize that the trans- regulation of regulatory SNPs on the transcript splicing of distal genes is preferentially associated with the regulation on the transcript splicing of the cis- located genes. More specific and intensive statistical analyses, as well as biological experiments, are required to test this hypothesis.

## Materials and Methods

### SNP data preprocessing

In the HapMap data (release 27), SNP genotypes are presented in a bi-allelic form, such as A/C. We transformed the genotypes of a SNP into numeric values by assigning an individual containing zero, one or two reference alleles with 0, 1 or 2. The undetermined genotypes of a SNP in individual cell lines were not computationally imputed. As for a specific SNP, the un-genotyped cell lines were excluded from the subsequent linear model analysis. We didn’t consider the SNPs (and genes) on mitochondrial chromosome in this study.

In particular, we employed the following procedures to select the ∼8000 SNPs (on chromosome one) used for model evaluation. (1) For each gene, the cis- located SNPs were organized into a subset; (2) The paired-wise composite linkage disequilibrium (LD) correlations among the SNPs within the subset were evaluated by t-tests, and the p-values were adjusted with Bonferroni method; and (3) The subset was refined so that the genotypes of selected SNPs are independent to each other with adjusted p-values (for the LD quantities) less than 0.01.

### RNA-seq data processing

We downloaded the RNA-seq read data from the NCBI SRA database (**Table 1**). The TopHat software [47] was employed to map the short reads onto the human genome (hg18) and the computationally identified exon-exon junctions. In the execution, we set "anchor length" as 4, and “--segment-length” as half of the read length. “mate-inner-dist” (for paired-end data) was estimated by the difference between the middle RNA fragment length and twice the read length. Other parameters were set as default in TopHat (v-1.3.2). The digital gene expression levels, i.e. FPKMs (Fragments Per Kilobase of exon model per Million mapped fragments), were estimated by Cufflinks (version 1.30) [48]. Exon-level gene expression profiling was inferred with a lab-owned R program. More specifically, by referring to the UCSC RefSeq gene table, we first counted the number of reads unambiguously mapped to the region of an exon. Then, the RPKM (Reads Per Kilobase of exon model per Million mapped reads) of an exon was calculated by the method documented in [49]. Finally, we calculated the rescaled RPKM (expression index) of an exon by dividing its RPKM with the average RPKM of the exons within the same RefSeq gene.

### Weighted linear mixed model (WLMM)

We assumed that cell lines represent the only random effect factor for gene expression traits. We also noted that the tested cell lines in the RNA-seq data sets are genetically independent of each other. Accordingly, the weighted linear mixed model [50], one of the variants of the traditional mixed model [51], [52], [53] under heteroscedasticity [54], for assessing the effects of fixed and random factors on an expression trait can be generally formulated as follows.



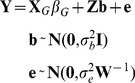
(1)


**Y** is an *r×1* vector containing the values of the expression trait, where, depending on the particular implementations, *r* may be the number of genotyped (for the cognate SNP) biological samples (n) or the multiplication of *n* by the number of exons (*n_e_)* in a gene (N). ***β*** is a *p×1* vector representing *p* fixed effect parameters. **b** is a *q×1* vector representing the random effect (SUBJECT) values of *q* genotyped cell lines. **X** and Z are *r×p* and *r×q* design matrices for fixed effects and random effects, respectively. **I** is a *q×q* identity matrix and **W** is an *r×r* diagonal matrix with the non-zero elements indicating the weights for the *n* observations.
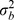
and 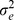 are the random effect variance component and residual variance component, respectively

### WLMM implementation for gene expression level analysis

The association between the expression level of a gene and the genotypes of a SNP was inferred by the following model, a specific form of (1). 
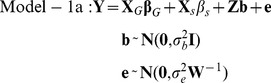
(2)


In (2), 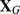 is an *n×4* design matrix for the fixed effect factor (GROUP) with respect to the four data sets involved in our study (**Table 1** **).** 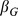
is a *4×1* vector indicating the effect parameters. 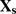
and 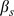 are the transformed SNP genotype vector and the regression coefficient, respectively.

When the correlations between samples are ignored, (2) can be simplified as the following heteroscedastic linear model (HLM) [54].
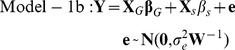
(3)


When both the variance heterogeneity of gene expression and the correlations between samples are ignored, (2) can be further simplified as the following ordinary linear model (OLM).
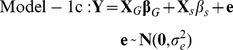
(4)


The analyses of Model-1a (and Model-2b presented in the next paragraph) were achieved by the *lmer* procedure included in the R package "lme4" [55]. REML (Residual Maximum likelihood) criterion was used to estimate the parameters. The weight matrix **W** was configured by the widely used strategy in the weighted least squares (WLS) [56] implementation for heteroscedastic linear models. That is, the assigned weight for an individual sample was inversely proportional to the variance of the gene expression levels of the group (data set) to which it belonged. Our main interest here was the significance of the regression coefficient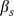. The null hypothesis to be tested is 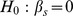 against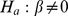 . From the *lmer* output, we could get the estimate of 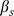 and its standard error but not the degree of freedom for the accordingly computed t statistic. Considering the number of freedom groups (cell lines) was large (>45), we adopted an empirical rule [57] and approximately inferred the p-value for the genotypic effect by a z-test. The simplified linear model analysis (including Model-1a, -1b, and Model-2b described in the following paragraph) was conducted by the *lm* procedure implemented in the R package "stats".

### WLMM implementation for transcript splicing analysis

We inferred the association between the genotypes of a SNP and the transcript splicing of a gene by the following weighted linear mixed model with the exon-level expression index (rescaled RPKM) as the dependent variable.
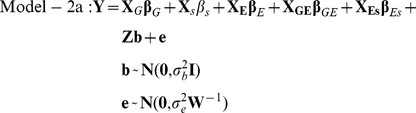
(5)


In (5), 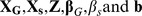 are defined similarly to (1) and (2) but the design matrices have *N* (rather than *n*) rows. EXON (representing exons) is included as another fixed factor with **X**_E_ (N×*n_e_)* and 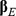
(*v*×1) as the design matrix and effect parameter vector, respectively. The term 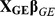 represents the interaction between the groups and exons. The term 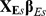
represents the interaction of the SNP genotypes and exons, by which the effect of the genotypes on transcript splicing was assessed in our study. Similar to [58], we tested the null hypothesis that there is no interaction (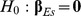) against 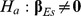.

When the correlations between samples are ignored, (5) can be simplified as the following heteroscedastic linear model.
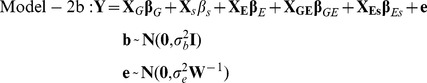
(6)


In the implementation, the assigned weight for an individual sample with respect to a specific exon was inversely proportional to the variance of the exon expression levels of the group (data set) to which it belongs. From the *lmer* output, we could get the F-statistic (*F*) for the term 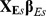
and the nominator degree (*v*_1_) of freedom. Because for most genes, the residual degree of the model was very large, we approximately assessed the p-value using a Chi-squared test. More specifically, we calculated the *X^2^* by *F×v_1_* [59] and determined the one-tail probability with respect to a Chi-squared distribution with *v_1_* as the degree of freedom.

The statistical analysis using Model-2a was computationally demanding, especially for the genes with over 60 exons, and sometimes (∼10% chances) failed to converge in the implementation, leading to a difficulty in controlling false discover rate (FDR). As a solution, we determined the significant associations between the transcript splicing of genes and the genotypes of SNPs by a joint analysis of Model-2a (with FDR<0.05) and Model-2b (with FDR<0.01).

### Estimation of FDR

We adapted the permutation-based method in [8] to estimate the false discovery rate (FDR) in the association analysis. For each SNP located within a gene or in the flanking sequences, we permuted the genotypes four times (for Model-1a and Model-2a) or ten times (for Model-2b), re-conducted the linear model analysis, and recorded the p-values for the effects of interest (See the section of WLMM implementation). By doing so, we established model-specific empirical null distributions for the p-values. We then compared a true distribution of p-values to the corresponding null distribution to estimate FDR. That is, we found a p-value threshold *z* such that 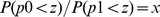, where *x* is the desired FDR, p0 is a p-value from the null distribution, p1 is a p-value from the true distribution, P(p0 < *z*) is the fraction of p-values from the permutations that fall below the threshold, and similarly, P(p1 < *z*) is the corresponding fraction in the non-permuted data.

### Visualization

Network modules in the case studies (**Figure 5**) was visualized by Cytoscape 2.8.1 [60].

## Supporting Information

Figure S1 **Double logarithmic charts for the numbers (x) of regulated target genes in the identified network paradigms.** C1: cisExpression_transExpression. C2: cisSplicing_transExpression. C3: cisExpression_transSplicing. C4: cisSplicing_transSplicing. P(X>x): the empirical cumulative distribution function. Most data points in C1-3 are adjacent to the (red) straight lines, indicating that the observed target numbers roughly fit a discrete power law distribution (α>1, x > = 1). In addressing C1 (C3) data, x was truncated with 150 (250) as the upper limit to alleviate the leverage effect of an extreme data point.
(PDF)

Table S1 **The associations between eQTL SNPs and the cis- located host genes.**
(XLSX)

Table S2 **The associations between sQTL SNPs and the cis- located host genes**.
(XLSX)

Table S3 **The summary of cisExpression_transExpression (C1) regulatory network modules.**
(XLSX)

Table S4 **The summary of cisSplicing_transExpression (C2) regulatory network modules.**
(XLSX)

Table S5 **The summary of cisExpression_transSplicing (C3) regulatory network modules.**
(XLSX)

Table S6 **The summary of cisSplicing_transSplicing (C4) regulatory network modules.**
(XLSX)
