# Supplementary material for: Inferring Polymorphism-Induced Regulatory Gene Networks Active in Human Lymphocyte Cell Lines by Weighted Linear Mixed Model Analysis of Multiple RNA-Seq Datasets
Source: PLoS One. 2013 Oct 30;8(10):e78868. doi: 10.1371/journal.pone.0078868 (PMC3813575; doi:10.1371/journal.pone.0078868)
Supplement: Figure S1 — Double logarithmic charts for the numbers (x) of regulated target genes in the identified network paradigms. C1: cisExpression_transExpression. C2: cisSplicing_transExpression. C3: cisExpression_transSplicing. C4: cisSplicing_transSplicing. P(X>x): the empirical cumulative distribution function. Most data points in C1-3 are adjacent to the (red) straight lines, indicating that the observed target numbers roughly fit a discrete power law distribution (α>1, x > = 1). In addressing C1 (C3) data, x was truncated with 150 (250) as the upper limit to alleviate the leverage effect of an extreme data point. (PDF) [file pone.0078868.s001.pdf]

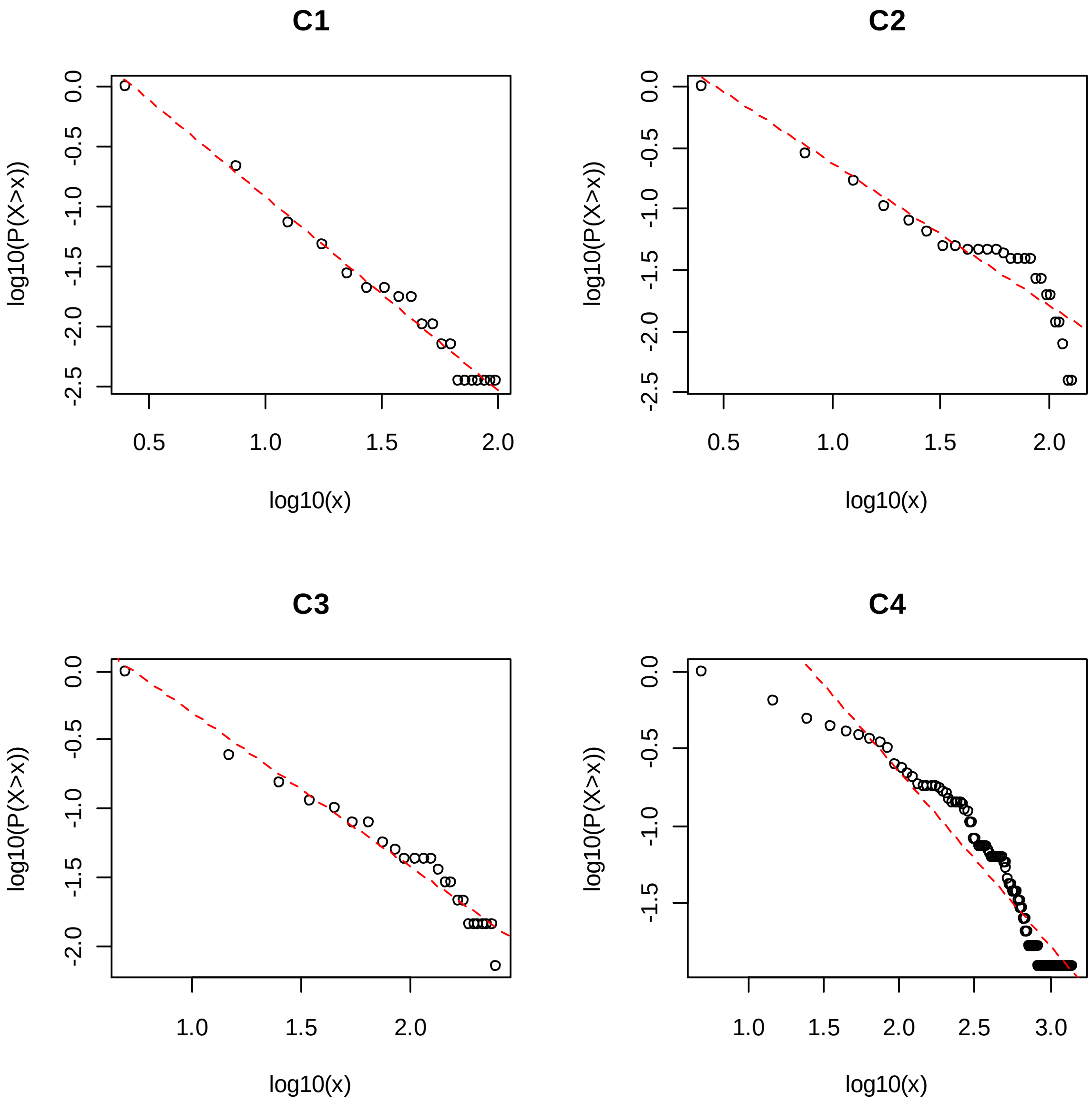

Figure S1: The double logarithmic charts for the numbers (x) of regulated target genes in the identified regulatory networks. **C1**: cisExpression_transExpression. **C2**: cisSplicing_transExpression. **C3**: cisExpression_transSplicing. **C4**: cisSplicing_transSplicing. P(X>x): the empirical cumulative distribution function. Most data points in **C1-3** are adjacent to the (red) straight lines, indicating that the observed target numbers roughly fit a discrete power law distribution $p(x) \propto x^{-\alpha}$ ($\alpha>1$, $x>=1$). In addressing C1 (C3) data, x was truncated with 150 (250) as the upper limit to alleviate the leverage effect of the extreme data point.
